# CT-Derived Patient-Specific Computer Simulation of the Novel Self-Expanding Evolut FX Implantation: A Case Series

**DOI:** 10.3390/jcm13206212

**Published:** 2024-10-18

**Authors:** Romy R. M. J. J. Hegeman, Simon E. van Putten, Leo Timmers, Benno J. W. M. Rensing, Uday Sonker, Severin Laengle, Martin Andreas, Martin J. Swaans, Jurriën M. ten Berg, Patrick Klein

**Affiliations:** 1 Department of Cardiothoracic Surgery, St. Antonius Hospital Nieuwegein, 3435 CM Nieuwegein, The Netherlands; 2 Department of Cardiothoracic Surgery, Amsterdam University Medical Center, 1105 AZ Amsterdam, The Netherlands; 3 Department of Cardiology, St. Antonius Hospital Nieuwegein, 3435 CM Nieuwegein, The Netherlands; 4 Department of Cardiac Surgery, Medical University of Vienna, 1090 Vienna, Austria; 5 Cardiovascular Research Institute Maastricht, 6229 HX Maastricht, The Netherlands

**Keywords:** aortic valve stenosis, TAVI, transcatheter aortic valve replacement, computer simulation, computer modeling, Evolut FX

## Abstract

**Background/Objectives**: Paravalvular leak and permanent pacemaker implantation remain relevant issues after transcatheter aortic valve implantation (TAVI). Novel device development as well as adequate preprocedural device selection can contribute to optimal outcomes. **Methods**: Computed tomography-based patient-specific computer anatomical analysis and simulation were used in addition to standard preprocedural preparation in three of the first Evolut FX cases in our center. Procedural and follow-up echocardiographic outcomes are presented. **Results**: Computed tomography-based computer simulation of Evolut FX resulted in implantation of a different size in one of three cases. In three cases of severe aortic valve stenosis, procedural as well as follow-up outcomes were satisfactory and in line with the simulated results of the chosen strategy. **Conclusions**: Preprocedural patient-specific computer simulation predicts and guides decision-making in TAVI with the Evolut FX platform. The combination of advanced preprocedural technology and novel technologies continues to contribute to the optimization of TAVI outcomes.

## 1. Introduction

Paravalvular leak (PVL) and permanent pacemaker implantation (PPI) remain the Achilles’ heel of transcatheter aortic valve implantation (TAVI), especially in self-expandable supra-annular valves [1]. In the past decade, technological advancements of the Evolut (Medtronic, Minneapolis, MN, USA) transcatheter heart valves (THV) have aimed to reduce these unfavorable procedural outcomes. Specifically, the improved sealing skirt design and implantation technique (i.e., with the use of the cusp-overlap view) of the Evolut PRO(+) THVs have contributed to reduced PVL and PPI rates as compared to its predecessors. However, achieving a relatively symmetrical and predictable final implant depth after release remains a challenge with the Evolut PRO+ system [1]. Especially in younger patients in whom lifetime management of both aortic valve and coronary disease becomes highly relevant, procedural optimization during the index TAVI procedure by achieving predictable implant depth and commissural alignment is becoming increasingly important [1]. In line with this, the newest-generation Evolut FX THV has undergone significant design changes to improve commissural alignment and achieve a more symmetrical deployment [1,2]. The novel single-spine delivery system facilitates delivery flexibility and trackability, whereas the newly added commissural gold markers that are located 3 mm from the inflow can provide guidance in achieving the right implant depth. Achieving a more symmetric THV implantation at the targeted implant depth has translated into optimized procedural outcomes. In two previous studies involving the Evolut FX system [1,3], mild or more PVL was shown to be reduced to 14–15%, compared to nearly 28–34% mild or more PVL with Evolut PRO in the US STS/ACC TVT registry and the Evolut PRO US Study [4,5].

Similarly, the implantation of the FX system also resulted in a lower PPI rate of 12% [1,3]. Although this was found to be an improvement over prior Evolut THV generations [4], this PPI rate remains relatively high. In addition to THV design improvements, optimization of procedural planning might contribute to further reducing the risk of unfavorable outcomes (i.e., PVL and PPI), such as with the use of computed tomography (CT) as the gold standard in prosthesis size selection [6]. CT-derived simulations of the valve implantation enable the prediction of PVL and the risk of conduction disorders on the basis of evaluation of the interaction between the TAVI device and the patient’s anatomy, allowing for a patient-tailored approach [7,8,9]. In this case series, we describe our first experience with the implantation of the Evolut FX system guided by CT-derived patient-specific implant simulation. As CT-derived simulations for Evolut FX are not yet available for clinical use, this series holds a distinctive position prior to its release.

## 2. Materials and Methods

FEops HEARTguide™ (FHG) (FEops, Ghent, Belgium) was used to make a step-by-step, patient-specific computer simulation of the TAVI procedure with the application of image segmentation and finite element analysis based on the available pre-operative CT images. The patient-specific computer model of the Evolut FX implantation provided an overview of multiple simulated Evolut FX sizes on different implantation depths, with the corresponding implant predictions regarding device and aortic root deformation, skirt apposition, potential PVL, and the contact pressure associated with conduction abnormalities. The results of the FHG were analyzed before the procedure by the TAVI operator(s) performing the procedure. On the basis of the FHG, the final choice of THV prosthesis, prosthesis size, and implant depth were determined.

## 3. Results

### 3.1. Case 1

**Figure 1:**
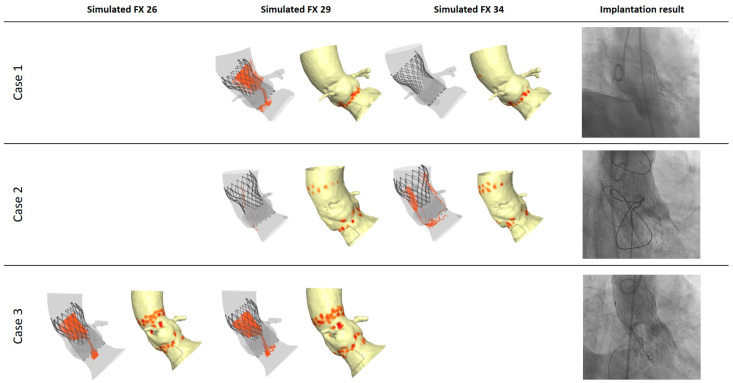
FHG simulation for the three cases of TAVI at high implantation depth with Evolut FX size 26 in the leftmost panel, Evolut FX size 29 in the middle-left panel, and Evolut FX size 34 in the middle-right panel. The rightmost panel shows procedural outcome on aortography.

An 81-year-old male with a history of posterior myocardial infarction treated with percutaneous coronary intervention of the right coronary artery (Table 1) presented with dyspnea and fatigue due to severe aortic stenosis (AoS) and was accepted for TAVI. After standard diagnostic work-up, the patient was scheduled to undergo transfemoral TAVI with implantation of a size 29 Evolut FX, as per routine CT-based anatomic measurements made with 3mensio Structural Heart (3mensio Medical Imaging B.V., Utrecht, The Netherlands) (Table 2). While echocardiography and 3mensio showed a tricuspid morphology, FHG anatomical analysis showed a bicuspid aortic valve with a calcified raphe between the right and non-coronary cups continuing into the left ventricular outflow tract. FHG simulation demonstrated improved skirt apposition with a reduction in PVL and a maintained low risk of conduction disturbances (CD) for size 34 compared with size 29 (Figure 1). Specific attention was paid to high deployment, as the simulated contact pressure index (CPI) was higher for placement at a medium implant depth compared with a high implant depth. The TAVI procedure was performed under local anesthesia via right transfemoral access. Predilatation was performed with a 22 mm True™ Dilatation Balloon (BD Interventional, Franklin Lakes, NJ, USA) under rapid pacing over the LV guidewire, after which an Evolut FX size 34 THV was implanted. Target implantation depth was acquired at 3 mm below the lower border of the non-coronary cusp (NCC) without re-sheathing. Post-implantation aortography showed satisfactory results with full frame expansion and no PVL, requiring no post-dilation. Postprocedural recovery was uneventful, and the patient was discharged on postprocedural day 1 without any conduction abnormalities. At the six-week follow-up, there were no conduction abnormalities, and transthoracic echocardiography (TTE) showed none to trace PVL.

### 3.2. Case 2

A 73-year-old female with a history of coronary artery bypass grafting and atrial fibrillation presented with dyspnea upon exertion as a result of severe AoS and was accepted for TAVI. FHG anatomic analysis confirmed conventional aortic valve measurements performed with 3mensio, and simulation predicted excellent outcomes for Evolut FX size 29 (Table 2 and Figure 1). No pre-dilation was performed, as anatomic analysis showed only moderate aortic valve calcification. The TAVI procedure was performed under local anesthesia via right transfemoral access. As primary deployment was performed at a planned implantation depth of 3 mm below the NCC with only trace PVL (as predicted) and without CD, there was no need for post-dilation. Postprocedural recovery was uneventful with discharge on postprocedural day 1. At the six-week follow-up, there were no conduction abnormalities, and TTE showed mild PVL.

### 3.3. Case 3

An 83-year-old female with a history of transient ischemic attack presented with congestive heart failure secondary to severe AoS and was accepted for TAVI. FHG anatomic analysis confirmed conventional aortic valve measurements made with 3mensio and simulation predicted excellent outcomes for Evolut FX size 29 (Table 2 and Figure 1). Pre-dilation was performed using a 23 mm True™ Dilatation Balloon. The TAVI procedure was performed under local anesthesia via right transfemoral access. Primary deployment was performed at a planned implantation depth of 3 mm below the NCC without the need for re-sheathing. Procedural success with only trace PVL and no conduction abnormalities was achieved without the need for post-dilation. Postprocedural recovery was uneventful, and the patient was discharged on postprocedural day 1 without any conduction abnormalities. At the six-week follow-up, TTE showed none to trace PVL, and there were no conduction abnormalities.

## 4. Discussion

Computer simulation of the implantation of the Evolut FX System with FEops HEARTguide™ is not yet available for clinical use, and in this case series, it is uniquely demonstrated before market release. Therefore, this case series gives a unique insight into the novel Evolut FX technology by showing its interaction with the anatomy of the aortic valve, root, and outflow tract in computer simulation. This case series shows adequately predicted PVL and CD in three cases of Evolut FX implantation after FHG simulation. Excellent procedural results at high implant depth were achieved in all three cases with no more than mild PVL at follow-up echocardiography and no permanent CD.

In the first-in-human multicenter study, the Evolut FX system showed a significant improvement over PRO+ in achieving commissural alignment, with fewer device recaptures and more symmetrical deployment [1]. Furthermore, improved procedural outcomes were observed in terms of PVL and PPI reduction in the first studies with the FX device [1,3]. Nevertheless, the reported incidences of especially mild PVL and new PPI remain higher compared with the outcome of surgical aortic valve replacement (SAVR) [10,11,12]. Minimizing the risk of these unfavorable outcomes is crucial to optimizing the outcome of TAVI with this self-expandable valve. This could be achieved not only by the development of novel valve technology but also through the optimization of procedural planning. Importantly, current standard procedural planning with CT-derived three-dimensional reconstructive imaging (e.g., 3mensio) lacks the ability to evaluate the device-host interaction required to predict the procedural outcome. Routine CT imaging combined with patient-specific computer modeling can, however, predict the interaction between the TAVI device and the patient’s unique anatomy, allowing for a detailed risk assessment of PVL and conduction disorders in advance of the procedure. By identifying patients with anatomical characteristics that predispose them to unfavorable outcomes, procedural execution may be optimized (i.e., by changing valve type, size, or implant depth). Previous cohort studies investigating the use of FHG in TAVI with self-expandable valves have shown that the selection of both valve size and target implant depth, as well as the TAVI execution to achieve the desired target depth, were influenced substantially for the sake of improved outcomes (i.e., reducing PVL and conduction abnormalities) [7,13]. Although the instruction for the use of Evolut FX recommends a depth of implantation at 3 mm below the NCC and left coronary cusp respectively (with a range of 1–5 mm), it is worth considering that the membranous septum length and location and grade of calcium that may prevent adequate sealing can differ by patient and can thus influence this ideal target depth. In line with this, valve morphology, leaflet calcification, and left ventricular outflow tract calcification can influence both the ideal valve depth as well as the ideal valve size for a specific patient. Potentially, the optimization of procedural planning by choosing the ideal valve size and implant depth by implementing a patient-tailored approach could in itself lead to fewer post-dilations and a decreased incidence of PVL and PPI.

## 5. Conclusions

This case series delivers an illustrative example of the combined strength of the improved THV technology of the Evolut FX system and advanced CT-based simulations. With the introduction of the new-generation Evolut FX THV, the incidence of any PVL and new PPM for the Medtronic THVs have been reported to be further reduced, yet they remain higher compared with SAVR. Potentially, the combination of the novel FX technology with advanced preprocedural imaging can help aim for perfection. This case series gives, before the market release of FHG use for Evolut FX, insight into procedural simulation of this new-generation valve. Further research is necessary to assess the added value of procedural implant simulation in reducing the PVL and PPI rate in TAVI with the Evolut FX system.

## Figures and Tables

**Table 1:** Baseline characteristics and medical history.

	Case 1	Case 2	Case 3
**Baseline characteristics**			
Age (years)	81	73	83
Gender	Male	Female	Female
Length (cm)	167	162	165
Weight (kg)	72.4	86.3	85
BMI (kg/m^2^)	26.0	32.9	31.2
NYHA class	Class III	Class III	Class III
EuroSCORE II (%)	2.85	4.95	3.66
**Baseline echocardiography**			
LVEF (%)	>50	40–49	>50
Max. pressure gradient (mmHg)	77	57	60
Mean pressure gradient (mmHg)	45	35	30
**Medical history**			
Hypertension	Yes	Yes	Yes
Diabetes	No	No	No
PAD	No	No	No
History of			
- PCI	Yes	No	No
- CABG	No	Yes	No
- AVR	No	No	No
- CVA/TIA	No	No	Yes, TIA

AVR, aortic valve replacement; BMI, body mass index; CABG, coronary artery bypass grafting; CVA, cerebrovascular accident; LVEF, left ventricular ejection fraction; NYHA, New York Heart Association; PAD, peripheral artery disease; PCI, percutaneous coronary intervention; TIA, transient ischemic attack.

**Table 2:** Anatomical characteristics and predictions.

	Case 1	Case 2	Case 3
**Anatomical characteristics—3mensio**			
Area (mm^2^)	487.1	507.3	442.6
Perimeter (mm)	79.6	80.7	77.7
Aorta minimum diameter (mm)	22.4	23.9	19.8
Aorta maximum diameter (mm)	27.3	27.2	28.5
Average diameter (mm)	24.8	25.6	24.1
Perimeter-based diameter (mm)	25.3	25.7	24.7
Area-derived diameter (mm)	24.9	25.4	23.7
Porcelain aorta	No	No	No
Membranous septum length (mm)	9.7	12.0	10.5
Calcium score (Agatston units)	2478	N.A.	1295
**Anatomical characteristics—FHG**			
Area (mm^2^)	460.5	492.3	459.5
Perimeter (mm)	77.0	79.3	76.7
Minimum diameter (mm)	22.6	24.5	22.4
Maximum diameter (mm)	26.9	26.9	26.1
Average diameter (mm)	24.8	25.7	24.3
Perimeter-based diameter (mm)	24.2	25.2	24.4
Area-derived diameter (mm)	24.5	25.0	24.2
Aortic valve morphology	Bicuspid	Tricuspid	Tricuspid
Grade of calcium	Severe	Moderate	Moderate
**Predicted outcomes—FHG**			
26H			
PVL (mL/s)	-	-	5.8
CPI (%)	-	-	12
26M			
PVL (mL/s)	-	-	5.0
CPI (%)	-	-	21
29H			
PVL (mL/s)	8.6	3.5	5.2
CPI (%)	5	1	5
29M			
PVL (mL/s)	10.7	0.1	3.3
CPI (%)	18	10	20
34H			
PVL (mL/s)	1.9	6.1	-
CPI (%)	2	0	-
34M			
PVL (mL/s)	2.7	0.0	-
CPI (%)	18	5	-

CPI, contact pressure index; FHG, FEops HEARTguide; N.A., not available; PVL, paravalvular leakage.

## Data Availability

The data presented in this study are available upon request from the corresponding author.

## References

[1] Zaid S., Attizzani G.F., Krishnamoorthy P., Yoon S.H., Palma Dallan L.A., Chetcuti S., Fukuhara S., Grossman P.M., Goel S.S., Atkins M.D. (2023). First-in-Human Multicenter Experience of the Newest Generation Supra-Annular Self-Expanding Evolut FX TAVR System. JACC Cardiovasc. Interv..

[2] Khera S., Krishnamoorthy P., Sharma S.K., Kini A.S., Dangas G.D., Goel S., Lerakis S., Anastasius M., Moreno P., Tang G.H.L. (2023). Improved Commissural Alignment in TAVR with the Newest Evolut FX Self-Expanding Supra-Annular Valve: First-in-Human Experience. JACC Cardiovasc. Interv..

[3] Merdler I., Case B., Bhogal S., Reddy P.K., Sawant V., Zhang C., Ali S., Ben-Dor I., Satler L.F., Rogers T. (2023). Early experience with the Evolut FX self-expanding valve vs. Evolut PRO+ for patients with aortic stenosis undergoing TAVR. Cardiovasc. Revascularization Med..

[4] Forrest J.K., Kaple R.K., Tang G.H.L., Yakubov S.J., Nazif T.M., Williams M.R., Zhang A., Popma J.J., Reardon M.J. (2020). Three Generations of Self-Expanding Transcatheter Aortic Valves: A Report from the STS/ACC TVT Registry. JACC Cardiovasc. Interv..

[5] Forrest J.K., Mangi A.A., Popma J.J., Khabbaz K., Reardon M.J., Kleiman N.S., Yakubov S.J., Watson D., Kodali S., George I. (2018). Early Outcomes with the Evolut PRO Repositionable Self-Expanding Transcatheter Aortic Valve with Pericardial Wrap. JACC Cardiovasc. Interv..

[6] Achenbach S., Delgado V., Hausleiter J., Schoenhagen P., Min J.K., Leipsic J.A. (2012). SCCT expert consensus document on computed tomography imaging before transcatheter aortic valve implantation (TAVI)/transcatheter aortic valve replacement (TAVR). J. Cardiovasc. Comput. Tomogr..

[7] El Faquir N., De Backer O., Bosmans J., Rudolph T., Buzzatti N., Bieliauskas G., Collas V., Wienemann H., Schiavi D., Cummins P. (2020). Patient-Specific Computer Simulation in TAVR with the Self-Expanding Evolut R Valve. JACC Cardiovasc. Interv..

[8] Dowling C., Firoozi S., Brecker S.J. (2020). First-in-Human Experience with Patient-Specific Computer Simulation of TAVR in Bicuspid Aortic Valve Morphology. JACC Cardiovasc. Interv..

[9] Hokken T., Van Mieghem N. (2022). CRT-700.26 Computed Tomography-Derived Predictive Simulations of Transcatheter Aortic Valve Replacement in Challenging Anatomies—The PRECISE TAVI Trial. JACC Cardiovasc. Interv..

[10] Fraccaro C., Tarantini G., Rosato S., Tellaroli P., D’Errigo P., Tamburino C., Onorati F., Ranucci M., Barbanti M., Grossi C. (2016). Early and Midterm Outcome of Propensity-Matched Intermediate-Risk Patients Aged ≥80 Years with Aortic Stenosis Undergoing Surgical or Transcatheter Aortic Valve Replacement (from the Italian Multicenter OBSERVANT Study). Am. J. Cardiol..

[11] Leon M.B., Mack M.J., Hahn R.T., Thourani V.H., Makkar R., Kodali S.K., Alu M.C., Madhavan M.V., Chau K.H., Russo M. (2021). Outcomes 2 Years After Transcatheter Aortic Valve Replacement in Patients at Low Surgical Risk. J. Am. Coll. Cardiol..

[12] Popma J.J., Deeb G.M., Yakubov S.J., Mumtaz M., Gada H., O’Hair D., Bajwa T., Heiser J.C., Merhi W., Kleiman N.S. (2019). Transcatheter Aortic-Valve Replacement with a Self-Expanding Valve in Low-Risk Patients. N. Engl. J. Med..

[13] El Faquir N., Rocatello G., Rahhab Z., Bosmans J., De Backer O., Van Mieghem N.M., Mortier P., de Jaegere P.P.T. (2020). Differences in clinical valve size selection and valve size selection for patient-specific computer simulation in transcatheter aortic valve replacement (TAVR): A retrospective multicenter analysis. Int. J. Cardiovasc. Imaging.

